# Predictive Efficacy of MiR-125b-5p, MiR-17-5p, and MiR-185-5p in Liver Metastasis and Chemotherapy Response Among Advanced Stage Colorectal Cancer Patients

**DOI:** 10.3389/fonc.2021.651380

**Published:** 2021-05-18

**Authors:** Daniel Sur, Loredana Balacescu, Simona S. Cainap, Simona Visan, Laura Pop, Claudia Burz, Andrei Havasi, Rares Buiga, Calin Cainap, Alexandru Irimie, Ovidiu Balacescu

**Affiliations:** ^1^ 11th Department of Medical Oncology, University of Medicine and Pharmacy “Iuliu Hatieganu”, Cluj-Napoca, Romania; ^2^ Department of Medical Oncology, The Oncology Institute “Prof. Dr. Ion Chiricuta”, Cluj-Napoca, Romania; ^3^ Department of Genetics, Genomics and Experimental Pathology, The Oncology Institute “Prof. Dr. Ion Chiricuta”, Cluj-Napoca, Romania; ^4^ Department of Pediatric Cardiology, Emergency County Hospital for Children, Pediatric Clinic no 2, Cluj-Napoca, Romania; ^5^ Department of Mother and Child, “Iuliu Hatieganu” University of Medicine and Pharmacy, Cluj-Napoca, Romania; ^6^ Research Center for Functional Genomics, Biomedicine and Translational Medicine, University of Medicine and Pharmacy “Iuliu Hatieganu”, Cluj-Napoca, Romania; ^7^ Department of Immunology and Allergology, University of Medicine and Pharmacy “Iuliu Hatieganu”, Cluj-Napoca, Romania; ^8^ Department of Pathology, The Oncology Institute “Prof. Dr. Ion Chiricuta”, Cluj-Napoca, Romania; ^9^ Department of Pathology, “Iuliu Hatieganu”, University of Medicine and Pharmacy, Cluj-Napoca, Romania; ^10^ 11th Department of Oncological Surgery and Gynecological Oncology, “Iuliu Hatieganu” University of Medicine and Pharmacy, Cluj-Napoca, Romania; ^11^ Department of Surgery, The Oncology Institute “Prof. Dr. Ion Chiricuta”, Cluj-Napoca, Romania

**Keywords:** biomarkers, exosomes, colorectal cancer, liver metastases, miR-125b-5p, miR-17-5p, miR-185-5p, chemotherapy response

## Abstract

MicroRNAs (miRNAs), a class of small non-coding RNAs represent potential biomarkers for colorectal cancer (CRC). The study hypothesized that miRNAs associated with liver metastases may also contribute to assessing treatment response when associated to plasma exosomes. In this study, we used two sets of biological samples, a collection of tumor tissues harvested from patients with CRC with and without liver metastases, and a collection of plasma from CRC patients with and without response to FOLFOX4/FOLFIRI regimens. We investigated 10 target miRNAs in the tissue of 28 CRC patients and identified miR-125b-5p, miR-17-5p, and miR-185-5p to be associated with liver metastasis. Further, we investigated the three miRNAs at the exosomal level in a plasma collection to test their association with chemotherapy response. Our data suggest that the elevated plasma levels of miR-17-5p and miR-185-5p could be predictive of treatment response. Overexpression of miR-17-5p and underexpression of miR-125b-5p and miR-185-5p in CRC tissue seem to be associated with metastatic potential. On the other hand, an increased expression of miR-125b-5p in plasma exosomes was potentially correlated with a more aggressive CRC phenotype.

## Introduction

Colorectal cancer (CRC) is a leading cause of cancer-related deaths worldwide (1). Poor prognosis and survival rates are mainly due to metastasis (2). Although the introduction of new cytotoxic and biologic therapies (anti-VEGF, anti-EGFR) has significantly improved patient prognosis, metastases to the liver and other distant organs impact clinical outcomes and quality of life (3).

MicroRNAs (miRNAs) are a class of small (19-24 nucleotides) non-coding RNA molecules that have the property of controlling gene expression post-transcriptionally (4). Due to their role in cancer progression and metastasis, miRNAs are considered an important class of molecules with the role of biomarkers. In CRC and other cancers, miRNAs are heavily dysregulated, affecting all cancer hallmarks (5).

MicroRNAs have also been associated with exosome trafficking from primary tumors to secondary metastatic sites (i.e., small extracellular vesicles associated with the metastatic process) (6, 7). Tumor-derived exosomes, through their cargo components, including miRNA and specific proteins, can alter the phenotype of the distant recipient cells by modulating their transcriptome in a Dicer-dependent manner (7). On this line, the exosomes released by CRC can transfer by organotropism, specific miRNAs to liver cells, sustaining the development of liver metastasis (8–10). Different miRNAs from tumor-related exosomes have been detected as biomarkers in the plasma of CRC patients (11, 12). A potential application of tissue or plasma miRNA detection would be to discriminate patients with or without metastases and stratification (or selection) of CRC patients for a given therapy. In a previous article, we pointed out the role of different miRNAs classes as candidates for CRC liver metastasis by modulating specific molecular pathways involved in liver metastasis of colorectal cancer (13). However, not all miRNAs altered in tumor tissue have the same correspondent alteration in the tumor-related exosomes. The level of miRNAs loaded into exosomes could be influenced by their mRNA-miRNA interaction in the donor cells (14). The mRNA-miRNA interactions inside a tumor cell can be affected by tumor progression and its response to the treatment. Thus, identifying tumor-exosomal miRNAs associated with both tumor invasion and metastasis and response to therapy is a major challenge in identifying new reliable markers.

Most studies investigating miRNAs as possible biomarkers are focused on either diagnosis, prognosis, or response to therapy. Identifying miRNA biomarkers to characterize a tumor phenotype better, predicting both metastasis and treatment response, represents a critical approach that we consider very challenging. Consequently, our study aims to identify miRNA biomarkers that could predict both liver metastasis and treatment response. In this regard, we have focused on identifying specific tissue miRNAs predicting CRC liver metastasis, which could also be associated with treatment response when evaluated in tumor-derived exosomes.

We focused on miR-17-5p (oncomiR) as a putative biomarker because previous studies indicated it as tissue and exosomal biomarker for liver metastasis. However, to our knowledge, there are no studies investigating the role of exosomal miR-17-5p as a treatment predictor for CRC liver metastasis treated with FOLFOX or FOLFIRI regimens. Nevertheless, recent data pointed out the association of high levels of miR-17-5p in primary tissue of CRC and its liver metastasis (15), an increased level of miR-17-5p in exosomes released by CRC with liver metastases (16, 17), and CRC-associated fibroblasts (18). Albeit the association of mir-17-5p with metastatic CRC resistance to therapy was previously investigated on cell lines, no study presents data on patients with CRC treated with FOLFOX or FOLFIRI (19).

Other miRNAs of interest that we focused on included two tumor suppressor miRNAs, miR-185-5p and miR-125b-5p. MiR-185-5p published data revealed its downregulation in CRC progression and metastasis (20), mediating metastasis and stemness when it is sponged by circRNAs (21) or inhibited by lncRNA (22). However, no studies present data about the association of exosomal-mir-185 with CRC liver metastasis, while only one study associated miR-185 expression of radiosensitivity in CRC (23). Downregulation of miR-125b-5p is associated with CRC proliferation (24) and prognosis (25). There is no data concerning exosomal miR-125b-5p role as a predictor for CRC liver metastasis. Nevertheless, aberrant downregulation of miR-125b-5p was recently associated with drug resistance in CRC (26) and liver metastasis (27).

The standard of care for advanced CRC is FOLFOX (i.e., leucovorin, 5-fluorouracil, and oxaliplatin) or FOLFIRI (i.e., leucovorin, 5-fluorouracil, and irinotecan) in conjunction with targeted therapies. Irinotecan and oxaliplatin are extensively used with 5-fluorouracil and leucovorin as the first-line and second-line treatments for metastatic CRC patients (28). The results of previous studies demonstrated that FOLFOX and FOLFIRI are standard regimens for novel clinical trials and can be used for most metastatic CRC patients (28, 29).

Our study aims to investigate if the miR-17-5p, and miR-185-5p and miR-125b-5p associated with liver metastases, may also contribute to the assessment of treatment response (using FOLFOX or FOLFIRI) when evaluated in plasma-derived exosomes.

## Methodology

### Patients and Samples

The present study was conducted on two CRC sample collections, including 28 FFPE tissues and 35 plasma samples, harvested from 63 patients with CRC, treated in The Oncology Institute Prof. Dr. Ion Chiricuta, Cluj-Napoca, Romania. The inclusion criteria were: 1) stages II-IV CRC diagnosis with radiological clinical progression in terms of liver metastasis, 2) availability of tumor tissue biopsies or surgical specimens, 3) availability of patients for blood sample collection, 4) HP confirmation of diagnosis by a pathologist, 5) measurability of the tumor according to the Response Evaluation Criteria in Solid Tumors criteria (version 1.1), 6) follow-up of at least 60 months from the first therapy for metastatic disease, 7) disease measurability at the baseline with demonstrated disease progression or response based on radiologic or clinical assessment.

The study was carried out based on the approval of the Ethics Committees of the Oncology Institute Prof. Dr. Ion Chiricuta and the University of Medicine and Pharmacy Iuliu Hatieganu, Cluj-Napoca, Romania. All patients gave their consent in accordance with the Declaration of Helsinki.

### Study Design

This study aims to identify miRNAs with a dual role in predicting liver metastasis and treatment response to FOLFOX4/FOLFIRI regimens in CRC. Because we have not a matching collection of tissue-plasma for the same patients, we proposed a study design based on retrospective and prospective approaches. The retrospective study included a group of FFPE samples harvested from CRC patients with and without liver metastasis at follow-up, while the prospective study included plasma harvested from CRC patients with different responses to FOLFOX4/FOLFIRI regimens. The clinical and demographic features were recorded for each patient.

#### Tissue Study

Our first aim was to identify specific miRNAs associated with CRC liver metastasis. In this part of the study, we included tissue samples from 28 patients with stage II or III CRC, with a very rigorous clinical follow-up. The patients were assigned into two groups: those who developed liver metastasis (M+) and those with no liver metastases (M-).

### Plasma Study

The second aim was to investigate if specific tissue miRNAs, associated with CRC liver metastasis, could predict CRC treatment response when they are evaluated in plasma exosomes. To this aim, a total of 35 patients with stage II-IV CRC were included in the study. Peripheral blood was collected before starting a FOLFOX4/FOLFIRI-based systemic therapy. The patients were divided into partial treatment responders (PR; n=16) and non-responders (NR; n=19).

### Treatment Schedule

None of the patients from the tissue group have been treated with radiotherapy or chemotherapy before surgery. The CRC tissue samples were obtained after CRC surgery. Subsequently, the patients received standard adjuvant chemotherapy based on fluoropyrimidine plus oxaliplatin.

All patients from the plasma group received systemic FOLFOX4/FOLFIRI chemotherapy. In the current study, 18 (51.42%) patients received the FOLFIRI treatment, and 17 (48.57%) subjects received FOLFOX4+/-Avastin, Cetuximab, or no targeted therapy, depending on their clinical features and mutational status, with 71.42% of the patients in this group undergoing surgery.

### Tissue Processing and RNA Extraction From FFPE Samples

From each FFPE tumor sample, five sections of 10 µm each were provided in similar conditions by macrodissection by a specialized pathologist. The RNA was isolated using a *miRNeasy FFPE kit (Qiagen*) according to the manufacture’s instructions. Shortly, the sections were deparaffinized and treated with Proteinase K as mentioned in the protocol. The recovered supernatant was further used for RNA extraction using silica gel columns, including DNase treatment. Finally, the total RNA was eluted with RNase-free water and stored at -80°C until its further use. The RNA quantification was evaluated by NanoDrop ND-1000 spectrophotometer (*Thermo Fisher Scientific*).

### Plasma Collection and RNA Extraction from Exosomes

Peripheral blood from CRC patients was collected at baseline by venipuncture in EDTA collection tubes. The plasma was separated by double centrifugation of blood at 4,000 and 12,000 rpm for 10 min at 4°C. Provided plasma was aliquoted (400 ul) and stored at -80°C until its further use. According to the producer’s instructions, about 400 ul of 0.8-µm-filtered plasma for each sample has been used for exosomes isolation with the Total Exosome Isolation Kit from plasma (Thermo Fisher). Shortly, the filtered plasma was treated with 0.05 volume of Proteinase K and incubated at 37°C for 10 minutes. About 120 ul of precipitation reagent was added to the sample, following a 30 minutes incubation at 4°C and a 10.000xg centrifugation. The resulting pellets (exosomes) were resuspended in 200 ul PBS and used for RNA extraction, with the *Total Exosome RNA and Protein Isolation Kit* (Thermo Fisher). In the extraction steps, before adding the Acid-Phenol-Chloroform as is mentioned in the protocol, we used a volume of 2.5 ul of exogenous cel-miR-39 spike-in (2x10^8^ transcripts), to be further used for normalization of miRNA expression.

### Selection of miRNAs of Interest

Our first thought to identify putative miRNA biomarkers that could have a double role in CRC liver metastasis and treatment response was to conduct an R&D study on a set of 84 miRNAs involved in modulating cancer stem cells status (data not presented). We performed a miScript miRNA PCR Array Human Cancer Stem Cells analysis (MIHS-118Z-Qiagen), considering that tumor stem cells are responsible for the invasive phenotype and treatment resistance (30). Our preliminary data indicated that miR-150-5p, miR-125b-5p, and miR-185-5p were statistically differentially expressed between CRC patients with and without liver metastasis. As a consequence, these three miRNAs were first considered for more extensive validation in FFPE tissue samples.

Further, based on the literature review, we selected several functionally relevant miRNAs based on two criteria: i) miRNAs associated with CRC liver metastases and ii) miRNAs mediating invasion and metastasis through exosomes (**Table 1**).

**Table 1:** MicroRNAs related to colorectal cancer liver metastasis.

No.	miRNAs	miRBase unique identifier	miRNA role	Ref
1	hsa-miR-17-5p	MIMAT0000070	oncomiR	(17)
2	hsa-miR-21-5p	MIMAT0000076	oncomiR	(31)
3	hsa-miR-92a-3p	MIMAT0000092	oncomiR	(17)
4	hsa-miR-31-5p	MIMAT0000089	oncomiR	(32)
5	hsa-miR-192-5p	MIMAT0000222	TS miR	(33)
6	hsa-miR-26a-5p	MIMAT0000082	TS miR	(34)
7	hsa-miR-150-5p	MIMAT0000451	TS miR	(35)
8	hsa-miR-125b-5p	MIMAT0000423	TS miR	(36)
9	hsa-miR-185-5p	MIMAT0000455	TS miR	(20)
10	hsa-miR-193a-3p	MIMAT0000459	TS miR	(37)
11	hsa-miR-16-5p	MIMAT0000069	TS miR (endogen normalizer for FFPE)	
12	cel-miR-39	MIMAT0000010	(Spike-in exogen normalization for exosomes) TS miR (endogen normalizer for FFPE)

hsa-miR, homo sapiens microRNA; oncomiR, oncogenic microRNA; TS miR, tumor suppressor microRNA.

In brief, we selected 3 miRNAs significantly expressed between CRC patients with and without liver metastasis in the pilot study (miR-150-5p, miR-125b-5p, and miR-185-5p) and other seven miRNAs of interest considering their role in cancer metastasis, exosomal presence, and treatment response. These miRNAs of interest, including oncomiRs [miR-17-5p (19), miR-21-5p (38), and miR-31-5p (39)] modulating the invasive phenotype of CRC, TS miRs [miR-125b-5p (40), miR-185-5p (41), and miR-193a-3p (42)] modulating tumor microenvironment, oncomiR [miR-92a-3p (17)], and TS miR [miR-192-5p (43), miR-26a-5p (44), and miR-150-5p (45)] mediating invasion and metastasis through exosomes. In addition, exogenous (cel-miR-39) and endogenous (miR-16-5p) controls were used for miRNA investigation. Only the miRNAs relevant in the tissue samples were further evaluated in plasma exosomes to identify putative tumor exosomal-miRNAs that may be associated with both tumor metastasis and treatment response.

### Assessment of microRNA Expression

To evaluate the level of miRNA expression, we used the one-step advanced miRNA system, through which we can simultaneously assess a large number of miRNAs using the same pre-amplified cDNA. In this line, we generated cDNAs from 10 ng of total RNAs extracted from each FFPE sample and 4 µl of exosomal RNAs extracted from each plasma sample, using *TaqMan^®^ Advanced miRNA cDNA Synthesis Kit* following the manufacturer’s protocol. Considering the low quantity of exosomal-miRNA in the plasma and the input limits for the cDNA synthesis kit, we maximized the volume of input RNA used for cDNA synthesis, using 4 µl of exosomal RNA of each sample. Finally, the cDNA was pre-amplified using universal RT miRNA primers.

A volume of 2 µl of diluted cDNA (1:10 v/v) was used to investigate miRNA expression with *TaqMan^®^ Fast Advanced Master Mix (2X)* and specific miRNA advanced assays, in a final volume of 10 µl, using the LC480 device (Roche) under specific miR-advanced PCR settings: 1) a step of removing RNA contaminants by activating the enzyme UNG (uracil-n glycosylase) at 55°C for 2 minutes; 2) amplifying the enzyme (Taq polymerase) at 95°C for 20 sec; 3) PCR amplification through a set of 40 PCR cycles including 2 reaction steps, at 95°C for 3 sec and at 60°C for 30 sec. The expression level of the miRNA of interest was calculated using the ΔΔCt relative quantification method. Considering its stable expression in a large number of biological samples, miR-16-5p was used to normalize the expression level of the investigated miRNAs in FFPE samples while cel-miR-39 was used to normalize the expression of exosomal miRNAs. MicroRNA expression was calculated using the ΔΔCt relative quantification method.

### Statistical Analysis

According to data distribution, the correlation between clinicopathological characteristics and tissue or plasma miRNAs expression was evaluated with Mann-Whitney U test or unpaired sample *t*-test for two categorical variables or Kruskall-Wallis test, followed by Dunn’s multiple comparison *post hoc* test in case of three categorical variables. The DFS was defined as the time from diagnosis until the appearance of the liver metastasis, censoring patients with no metastasis at their last follow-up. In order to evaluate the association between DFS and miRNAs, each miRNA expression was classified according to its median value, based on which the patients were allocated into high- or low-expression group. The survival curves were estimated using the Kaplan-Meier method, and log-rank test was used to compare the survival distributions. Multivariate Cox proportional hazards regression models were used to adjust for age, gender and tumor grade.

## Results

### Patients and Tumor Characteristics

The baseline characteristics of the patients enrolled in the study are detailed in **Table 2**. There were no significant differences between the tissue and plasma datasets in terms of the distribution of pathological and other characteristics, except for tumor size. In this regard, more patients with T3 tumors were included in the tissue study, while those with T3 and T4 tumors were evenly distributed in the plasma study. Considering the lymph nodes status, N1 and N2 were more frequently observed in both tissue and plasma investigations. The G2 (moderate differentiated) status was more frequent in both study groups. Regarding tumor location, for the tissue samples, we included 17 left-sided CRC samples and 11 right-sided CRC tumors, while plasma samples were collected from 24 patients that had left-sided CRC tumors and 11 patients who presented right-sided CRC tumors. All the samples evaluated in the study were adenocarcinomas (Adk).

**Table 2:** Clinical and pathological data of the patients enrolled in tissue and plasma study.

Variable	Tissue study (n=28)	Plasma study (n=35)	P-value	Statistical test
	*Median (range)*	*Median (range)*		
**Age (years)**	55.5 (36-84)	60 (19-82)	0.598	Mann-Whitney U test
**Body mass index**	25.6 (17.3-36.5)	25.7 (18.4-40.4)	0.682	Mann-Whitney U test
	***n (%)***	***n (%)***		
**Gender**				
F	12 (42.9%)	13 (37.1%)	0.796	Fischer’s exact test
M	16 (57.1%)	22 (62.9%)		
**Tumor size**				
T2	1 (3.6%)	1 (2.9%)	0.0174*	Fischer’s exact test
T3	22 (78.6%)	16 (45.7%)		
T4	5 (17.9%)	16 (45.7%)		
NA	–	2 (5.7%)		
**Lymph nodes**				
N0	3 (10.7%)	6 (17.1%)	0.566	Fischer’s exact test
N1	14 (50.0%)	12 (34.3%)		
N2	11 (39.3%)	13 (37.1%)		
Nx	–	4 (11.4%)		
**Metastasis**	***In evolution***	***Baseline***		
M-/M0	16 (57.1%)	11 (31.4%)	–	–
M+/M1	12 (42.9%)	22 (62.9%)		
Mx	–	2 (5.7%)		
**Lymphatic invasion**				
L0	8 (28.6%)	7 (20.0%)	0.999	Fischer’s exact test
L1	18 (64.3%)	15 (42.9%)		
Lx	2 (7.1%)	13 (37.1%)		
**Perineural invasion**				
P0	16 (57.1%)	11 (31.4%)	0.524	Fischer’s exact test
P1	7 (25.0%)	8 (22.9%)		
Px	5 (17.9%)	16 (45.7%)		
**Grade**				
G1	5 (17.9%)	4 (11.4%)	0.926	Fischer’s exact test
G2	18 (64.3%)	19 (54.3%)		
G3	5 (17.9%)	5 (14.3%)		
NA	–	7 (20.0%)		
**Stage**				
S2	3 (10.7%)	4 (11.4%)	0.168	Fischer’s exact test
S3	25 (89.3%)	8 (22.9%)		
S4	–	23 (65.7%)		

*p≤0.05.

### Investigation of MicroRNA in Colorectal Cancer Tissue

Liver metastasis is the leading cause of death among CRC patients. Based on the evidence, approximately 50% of CRC patients develop liver metastases (46). MicroRNAs are involved in the post-transcriptional regulation of mRNAs and are abnormally expressed in cancer with an essential role in tumorigenesis. As a comprehensive analysis of the combined effects of altered activities of miRNAs in CRC has not been carried out, there are still many questions why miRNAs might affect tumor progression or patient outcomes (11).

**Figure 1:**
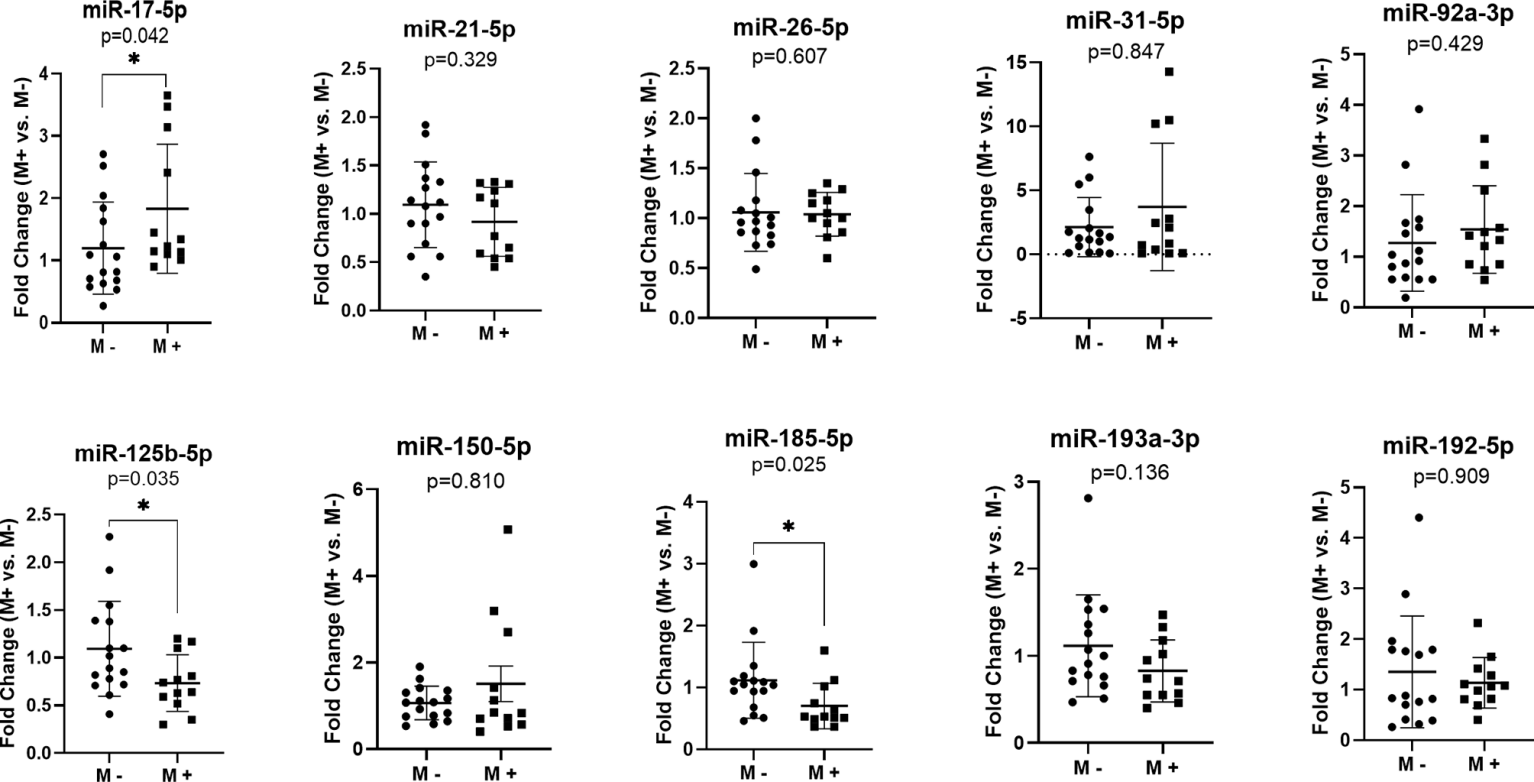
MicroRNAs expression in the primary tumor tissues of colorectal patients with liver metastases (M+) (n=12) *versus* patients without metastases (M-) (n=16); according to data distribution, differential expression was tested using the non-parametric Mann-Whitney U test (miR-17-5p; miR-21-5p; miR-26-5p; miR-31-5p; miR-92a-3p; miR-150-5p; miR-185-5p; miR-193a-3p; miR-192-5p) or parametric unpaired sample t-test (miR-125b-5p). Fold change for each sample was calculated relative to M- group. *p≤0.05.

In order to identify specific miRNA associated with liver CRC metastasis, we investigated the expression of 10 target miRNAs in the FFPE tissue of the primary tumors of CRC patients with and without liver metastases. Our results indicated an increased level of miR-17-5p (Fold change (FC)=1.83, p=0.042) in the liver metastases group (M+) as compared to that in non-metastatic (M-). Furthermore, a down-regulation of miR-125b-5p (FC=0.73, p=0.035) and miR-185-5p (FC=0.70, p=0.025) was observed in M+ patients compared to M- patients (**Figure 1**).

We performed the analysis of the studied microRNAs in the following publicly available colorectal cancer datasets: GSE35834, GSE147603, GSE81581, GSE98406, GSE56350, GSE53159, GSE51429 and GSE46622. In GSE56350 dataset we found a decreased expression of hsa-miR-185-5p (FR=-1.87, p=0.0004, FDR 2%) in CRC liver metastases (n=15) compared to primary tumors (n=46). Analysis of 14 matched CRC liver metastases and primary tumors (GSE98406 dataset) showed downregulation of hsa**-**miR-125-5p (FR=-2.07, p=0.034, FDR 79%) while expression profiling by RT-PCR in the GSE51429 training set highlighted a slight under-expression (FR=-1.11, p=0.011, FDR 12%) in CRC liver metastasis (n=12) vs. primary tumors (n=20). Making use of GSE81581 data, we evaluated hsa-miR-17-5p expression in 19 CRC liver metastases versus 23 primary tumors. The results pointed out a 1.3-fold increase in expression of has-miR-17-5p in liver metastases tissues (FR=1.32, p=0.049, FDR 23%).

We also analyzed the correlations among the three miRNAs expression and the clinicopathological data (**Table 3**). Our data revealed that high miR-17-5p expression was significantly correlated with tumor size (FC T4 *vs.* T3 = 2.01, p=0.027) and perineural invasion (FC P1 *vs.* P0 = 1.67, p=0.01). However, miR-125b-5p and miR-185-5p showed no significant correlation with the clinicopathological data except being correlated to metastases.

**Table 3:** Relation of clinicopathological data and miRNA expression while comparing patients with and without metastases in the tissue study.

Variable	miR-17-5p P-value	miR-125b-5p P-value	miR-185-5p P-value	Statistical test
**Age** <50 *vs.* >50	0.718	0.631	0.362	Mann Whitney
**Gender** M *vs.* F	0.099	0.296	0.331	Mann Whitney
**Tumor size** T3 *vs.* T4	0.027*	0.595	0.973	Mann Whitney
**Lymph nodes** N0 *vs.* N1 *vs.* N2	0.064	0.931	0.268	Kruskal-Wallis
**Metastasis (in evolution)** M- *vs.* M+	0.042*	0.035*	0.025*	Mann Whitney
**Lymphatic invasion** L0 *vs.* L1	0.304	0.597	0.907	Mann Whithey
**Perineural invasion** P0 *vs.* P1	0.010*	0.547	0.508	Mann Whitney
**Grade** G1 *vs.* G2 *vs.* G3	0.986	0.981	0.646	Kruskal-Wallis
**Stage** S2 *vs.* S3	0.710	0.766	0.188	Mann Whitney

*p≤0.05.

**Figure 2:**
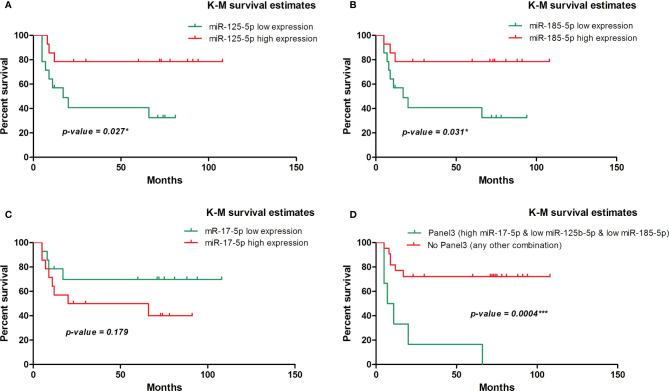
Kaplan-Meier survival curve for disease-free survival in 28 patients with colorectal cancer involved in the tissue study. **(A)** According to dichotomized miR-125-5p expression, (HR=3.7, 95% CI=1.162-11.81, p=0.027* [log-rank test]). **(B)** According to dichotomized miR-185-5p expression, (HR=3.59, 95% CI=1.127-11.41, p=0.031* [log-rank test]). **(C)** According to dichotomized miR-17-5p expression, (HR=0.45, 95% CI=0.144-1.435, p=0.179 [log-rank test]). **(D)** According to dichotomized expression pattern of miR-17-5p, miR-125b-5p and miR-185-5p observed in CRC tissue (HR=19.43, 95% CI= 3.772 - 100.1, p= 0.0004*** [log-rank test]).

We further evaluated the association of miRNAs expression with disease-free survival (DFS) of 28 FOLFOX4/FOLFIRI CRC treated patients. At the time of evaluation, the median follow-up was 26.5 months. In the univariate survival analysis, miR-125b-5p (HR=3.7, 95% CI=1.162-11.81, p=0.027) and miR-185-5p (HR=3.59, 95% CI=1.127-11.41, p=0.031) were significantly associated with DFS, the log-rank tests pointed out that CRC patients with low tissue levels of miR-125-5p and miR-185-5p had significantly shorter DFS than those with high tissue levels of these miRNAs (**Figures 2A, B**). In the multivariate survival analysis, miR-125b-5p was an independent prognostic factor for DFS (HR=4.971, 95% CI=1.12-22.067, p=0.035, **Table 4**, Section A) but miR-185-5p did not reach the significance threshold, although it had a low p-value (HR=3.834, 95% CI=0.976-15.062, p=0.054, **Table 4**, Section B). MiR-17-5p had no prognostic value either in univariate (**Figure 2C**) or multivariate analysis (**Table 4**, Section C). Further, we tested if there is a combined effect of these 3 microRNAs. We found that the patients with high tissue expression of miR-17-5p and low tissue expression of miR-125b-5p and miR-185-5p, assigned to group Panel 3, had shorter DFS than patients with any other combination (HR=19.43, 95% CI= 3.772-100.1, p= 0.0004) as resulted from log-rank univariate analysis (**Figure 2D**) and multivariate survival analysis (HR=6.116, 95% CI=1.714-21.827, p=0.005, **Table 4**, Section D). When we considered combined effect of these microRNAs regardless of the observed pattern in tissue, none of them had a prognostic value. (**Table 4**, Section E).

**Table 4:** Multivariate survival analysis of (Section A) miR-125b-5p; (Section B) miR-185-5p; (Section C) miR-17-5p; (Section D) Panel3 expression pattern (high tissue miR-17-5p expression & low tissue miR-125b-5p expression & low tissue miR-185-5p expression); (Section E) combined effect of miR-17-5p, miR-125b-5p and miR-185-5p adjusted for age, gender and tumor grade.

** Section A**			
**Variable**	**HR**	**95% CI for HR**	**p-value**
Gender (M *vs.* F)	0.766	0.21-2.794	0.686
Age	0.988	0.933-1.046	0.671
Tumor grade (G3 *vs.* G2 *vs.* G1)	3.04	0.737-12.542	0.124
miR-125b-5p (low *vs.* high)	4.971	1.12-22.067	0.035*
**Section B**			
**Variable**	**HR**	**95% CI for HR**	**p-value**
Gender (M *vs.* F)	0.575	0.142-2.336	0.439
Age	1.02	0.956-1.088	0.551
Tumor grade (G3 *vs.* G2 *vs.* G1)	1.886	0.699-5.093	0.21
miR-185-5p (low *vs.* high)	3.834	0.976-15.062	0.054
**Section C**			
**Variable**	**HR**	**95% CI for HR**	**p-value**
Gender (M *vs.* F)	0.634	0.137-2.927	0.56
Age	1.003	0.938-1.072	0.932
Tumor grade (G3 *vs.* G2 *vs.* G1)	2.041	0.739-5.632	0.168
miR-17-5p (low *vs.* high)	0.423	0.121-1.474	0.177
**Section D**			
**Variable**	**HR**	**95% CI for HR**	**p-value**
Gender (M *vs.* F)	1.037	0.225-4.772	0.963
Age	0.992	0.929-1.06	0.818
Tumor grade (G3 *vs.* G2 *vs.* G1)	2.461	0.691-8.767	0.165
Panel3 (Panel3 *vs.* No Panel3)	6.116	1.714-21.827	0.005**
**Section E**			
**Variable**	**HR**	**95% CI for HR**	**p-value**
Gender (M *vs.* F)	0.999	0.227-4.407	0.999
Age	0.994	0.93-1.062	0.85
Tumor grade (G3 *vs.* G2 *vs.* G1)	3.414	0.819-14.234	0.092
miR-17-5p (low *vs.* high)	0.364	0.096-1.387	0.139
miR-125b-5p (low *vs.* high)	3.518	0.604-20.488	0.162
miR-185-5p (low *vs.* high)	2.133	0.415-10.953	0.364

Hazard ratio (HR) were obtained from Cox proportional hazards regression models, HR>1 indicating that the low expression of miRNAs or Panel3 expression pattern is associated with short DFS. *p≤0.05; **p≤0.01.

### Investigation of MicroRNA in Plasma Exosomes

Exosomes are being involved in the metastatic process of a primary tumor transferring microRNA cancer-specific cargo. Based on the evidence, they can be identified in the plasma of patients (47). In line with this, we investigated if the liver metastasis-associated exosomal miRNAs could predict the treatment response in CRC patients. The miRNAs that were differentially expressed in liver metastases group *vs.* non-metastatic one (i.e., miR-17-5p, miR-125b-5p, miR-185-5p) and had a prognostic value (i.e., miR-125b-5p and miR-185-5p) in tissue study were evaluated in the plasma exosomes of CRC patients with partial treatment responses (PR) or non-responses (NR) including progressive and stationary disease. Treatment response included CT/PET-CT assessment according to clinical evaluation protocols.

**Figure 3:**
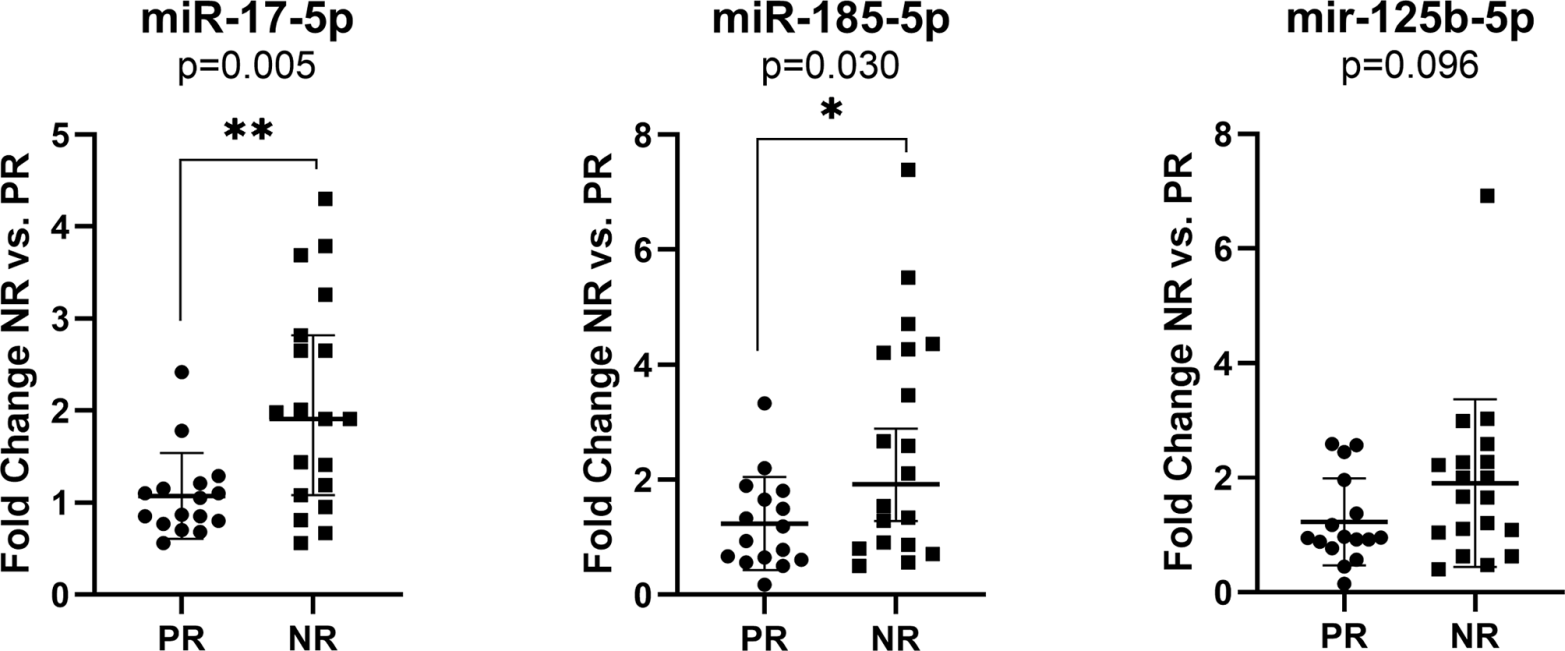
Expression of miR-17-5p, miR-185-5p, and miR-125b-5p in the plasma exosomes of non-responders *versus* responder (n=19/16) colorectal cancer patients regardless of the treatment received. According to data distribution, differential expression was tested using the non-parametric Mann-Whitney U test. Fold change for each sample was calculated relative to PR group. *p≤0.05; **p≤0.01.

The exosomal miR-17-5p (FC NR vs. PR=1.92, p=0.005) and miR-185-5p (FC=2.12, p=0.030) expression were significantly upregulated in nonresponder patients compared to patients with complete or partial response to therapy, regardless of the treatment received (**Figure 3**).

**Figure 4:**
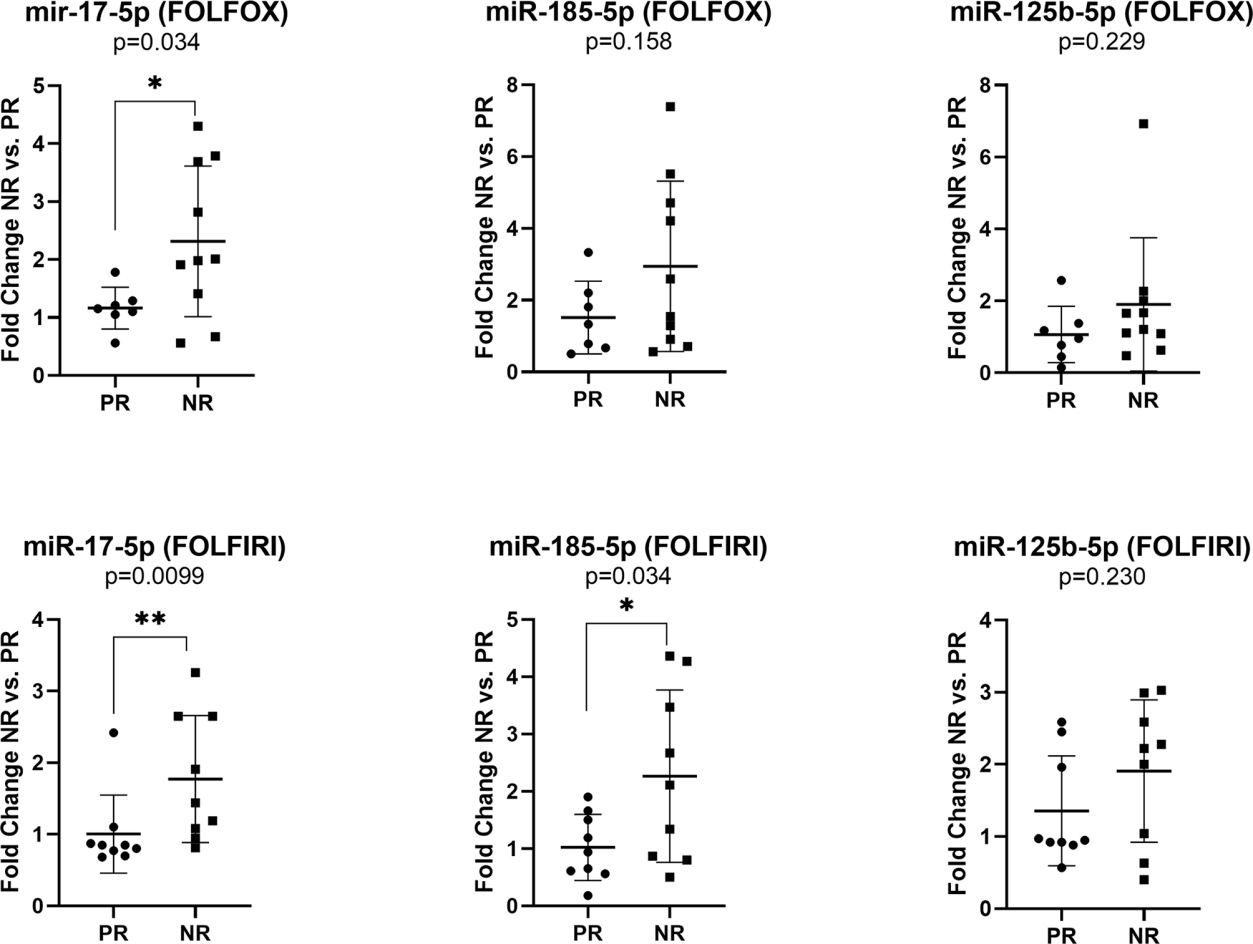
Expression of miR-17-5p, miR-185-5p, and miR-125b-5p in the plasma exosomes of non-responders *versus* responder colorectal cancer patients for FOLFOX4 (n=10/7) and FOLFIRI (n=9/9) therapy. According to data distribution, differential expression was tested using the non-parametric Mann-Whitney U test (miR-17-5p and miR-125b-5p) or parametric unpaired t-test (miR-185-5p). Fold change for each sample was calculated relative to PR group *p≤0.05; **p≤0.01.

The expression of miR-17-5p was significantly increased in non-responders versus responders when considering both FOLFOX4 (FC NR *vs.* PR=1.99, p=0.034) or FOLFIRI (FC NR *vs.* PR=1.76, p=0.0099) treatment. On the other hand, high expression of miR-185-5p (FC NR *vs.* PR=2.22, p=0.034) was statistically correlated with a lack of response only for FOLFIRI therapy (**Figure 4**).

The results revealed no statistical significance for miR-125b-5p exosomal expression in terms of treatment response. However, analyses between miR-125b-5p plasma exosomes and clinicopathological data revealed an increased expression of miR-125b-5p in the plasma of patients with high tumor grade (FC G3 *vs.* G2 = 1.73, p=0.016) and stage (FC S4 *vs.* S3 = 2.04, p=0.046; (**Table 5**).

**Table 5:** Significant differences between miRNAs with clinical data in the studied plasma samples.

Variable	miR-17-5p P-value	miR-125b-5p P-value	miR-185-5p P-value	Statistical test
**Age** <50 vs. >50	0.418	0.324	0.809	Mann Whitney
**Gender** M *vs.* F	0.891	0.707	0.932	Mann Whitney
**Tumor size** T3 *vs.* T4	0.157	0.146	0.317	Mann Whitney
**Lymph nodes** N0 *vs.* N1 *vs.*N2	0.329	0.367	0.371	Kruskal-Wallis
**Metastasis (baseline)** M0 *vs.* M1	0.339	0.163	0.479	Mann Whitney
**Lymphatic invasion** L0 *vs.* L1	0.778	0.481	0.887	Mann Whitney
**Perineural invasion** P0 *vs.* P1	0.772	0.265	0.710	Mann Whitney
**Grade** G1 *vs.* G2 *vs.* G3	0.500	0.016* **(G2 *vs.*G3)**	0.393	Kruskal-Wallis
**Stage** S2 *vs.* S3 *vs.* S4	0.396	0.046* **(S3 *vs.* S4)**	0.950	Kruskal-Wallis

*p≤0.05.

The summary of the two studies is presented in **Table 6**. Our data suggest that the elevated plasma levels of miR-17-5p and miR-185-5p could be predictive of treatment response. Overexpression of miR-17-5p and underexpression of miR-125b-5p and miR-185-5p in CRC tissue seem to be associated with metastatic potential. On the other hand, an increased expression of miR-125b-5p in plasma exosomes was potentially correlated with a more aggressive CRC phenotype.

**Table 6:** Summary of the tissue and plasma studies.

	miR-17-5p expression	miR-125b-5p expression	miR-185-5p expression
**Tissue study**			
*M+ vs. M-*	↑	↓	↓
*T3 vs. T2*	↑		
*P1 vs. P0*	↑		
*Univariate Survival Analysis*		↓ - short DFS	↓ - short DFS
*Multivariate Survival Analysis*		↓ - short DFS	
**Plasma study**			
*NR vs. PR (FOLFOX4 & FOLFIRI)*	↑		↑
*NR vs. PR (FOLFOX4)*	↑		
*NR vs. PR (FOLFIRI)*	↑		↑
G3 *vs.* G2		↑	
S4 *vs.* S3		↑	

## Discussion

The results of our study revealed that tissue expression of miR-17-5p, miR-125b-5p, and miR-185-5p is correlated with CRC liver metastasis. Also, in the plasma samples, exosomal miR-17-5p and miR-185-5p were predictive for treatment response. Accordingly, these miRNAs could act as a starting point for identifying new predictive biomarkers. MicroRNAs play essential roles in tumorigenesis and metastasis processes; thus, they could be used as prognostic and therapy predictive measures in various cancers (48, 49).

MiR-17-5p plays the role of oncomiR in CRC cases (50), while miR-125b-5p and miR-185-5p act as tumor suppressors (51, 52). First, our results from the tissue study indicated that high levels of miR-17-5p in primary CRC could be associated with metastatic potential and serve as a predictive biomarker. Furthermore, miR-17-5p appeared over-expressed in the group of patients with liver metastases, suggesting a possible involvement in the metastasis process. On this line, several studies confirmed the overexpression of miR-17-5p in CRC tissue specimens (53–55). However, the overexpression of miR-17-5p could be due to the interaction with the tumor microenvironment or due to liver metastasis in CRC. Therefore, the perception of miR-17-5p pathways could help in explaining the mechanisms of principal liver metastases in CRC (15).

On the other hand, miR-185-5p and miR-125b-5p appear down-regulated in CRC tissue compared to normal mucosa in other existing studies (52, 56). These results are similar to ours, implying that miR-185-5p and miR-125b-5p could bring new information for discriminating and stratifying CRC patients. Our results also showed that patients with low tissue expression of miR-125b-5p and miR-185-5p has a shorter DFS compared to patients with elevated tissue levels of these miRNAs. The univariate/multivariate analysis showed that miR-17-5p had no prognostic feature. Unfortunately, there aren’t any large-scale studies focusing on these two miRNAs in the literature concerning CRC for further underlying this position.

Second, our prospective study, including plasma samples from a cohort of 35 patients with advanced CRC, showed that overexpressed exosomal miR-17-5p and miR-185-5p were associated with treatment response. Exosomal miR-125b-5p had no statistical significance concerning treatment response. There are scarce data about the association of exosomal miRNAs with prognosis and treatment response in CRC cases, although recent studies have suggested that miRNA from the tumor or plasma exosomes could serve as cancer biomarkers to assist in prognosis and prediction of treatment responses (57, 58). Previous studies have demonstrated the potentiality of exosomal miR-17-5p as a non-invasive biomarker for both diagnosis and predicting tumor grade and stage in CRC (17, 59). Moreover, exosomal miR-17-5p and miR-185-5p are significantly upregulated in non-responsive patients treated with FOLFOX4/FOLFIRI (60, 61).

In patients with metastatic CRC, two miRNAs (i.e., miR-107 and miR-99a-3p) were found to predict response to traditional chemotherapy treatment (62). In addition, miR-224 and miR-143 were associated with treatment response to fluoropyrimidine-based chemotherapy (63). Serum miR-19a appears upregulated in advanced CRC cases that manifest resistance to FOLFOX, suggesting its potential to evaluate the treatment response (64). In another study, high expression of miR-625-3p was linked to poor response to FOLFOX/XELOX (Oxaliplatin and capecitabine) regimen in patients with metastatic CRC (65). Plasma exosomal miR-125b can be used as an indicator of resistance to mFOLFOX6 chemotherapy in patients with advanced or recurrent CRC (66). On the other hand, exosomal miR-128-3p can be used as a biomarker and also a therapeutic tool to improve the response to oxaliplatin in advanced CRC patients with acquired resistance to this drug (67). In rectal cancer, the profiling signature of miR-17/92 cluster can provide a reliable biomarker source for treatment response (68).

Our results showed that miR-17-5p was highly expressed in the plasma exosomes of non-responsive CRC patients regardless of the received treatment schedule (69). Although, the lack of association of miR-17-5p with DFS is surprising in the context of its differential expression in liver metastases versus non-metastatic patients, this result could be related to the size of the study group. In an *in-vitro* study, chemotherapy seemed to increase the expression of miR-17-5p in CRC cells and induce chemoresistance by repressing the PTEN factor (19). A panel of six miRNAs, which included miR-17-5p, proved to have biomarker potential in predicting response to 5-FU-based chemotherapy in metastatic first-line CRC patients (70).

Based on the evidence, miR-125a and miR-125b are underexpressed in the tumoral tissues of CRC, compared with adjacent nontumoral tissue (71). A more recent study on CRC cell lines showed that miR-125 targets and inhibits vascular endothelial growth factor (VEGF) expression and can be used as a therapeutic target (72). In our study, the prognostic role of miR-125b-5p and miR-185-5p was correlated with a significantly shorter DFS. It is also known that miR-185 acts as a tumor suppressor in other gastrointestinal tumors like gastric cancers (73) and hepatocellular carcinomas (74).

In an experimental study, Dong-xu W (20). showed that miR-185 overexpression acts as a tumor suppressor in CRC cells by suppressing the Wnt/β-catenin signal. High expression of miR-185 and low expression of miR-133b are correlated with poor survival and metastasis in CRC cases (41). A previous study demonstrated the role of STIM1 in the metastasis of CRC and that miR-185 directly targets the STIM1 pathway, making it a potential biomarker (75).

The small number of used samples gives the limitation of our study. However, considering our study’s design with a statistical power limited for clinical application, our data can be regarded as hypothesis-generating for future studies.

## Conclusion

The results of the current study revealed that the investigated miRNAs (i.e., miR-17-5p, mir-125b-5p, and miR-185-5p) significantly correlated with metastasis and treatment response of advanced CRC patients. These miRNAs can act as great non-invasive biomarkers for the prediction of CRC patients at high risk of liver metastasis and treatment evaluation for CRC subjects. However, our data should be validated on larger cohorts of patients to ensure the efficacy of these miRNAs in predicting both metastasis and treatment failure in advanced CRC.

## Data Availability Statement

The raw data supporting the conclusions of this article will be made available by the authors, without undue reservation.

## Ethics Statement

The studies involving human participants were reviewed and approved by Ethics Committees of the Oncology Institute Prof. Dr. Ion Chiricuta and the University of Medicine and Pharmacy Iuliu Hatieganu, Cluj-Napoca, Romania. The patients/participants provided their written informed consent to participate in this study.

## Author Contributions

Conceptualization: OB, DS and LB. Methodology: OB, LB and DS. Software: OB and LB. Validation: SV, LP, LB, and OB. Formal analysis: OB, LB, SC. Investigation: DS, CB, SC, SV, LP, and RB. Resources: OB, CC, SC, and RB. Data curation: SV, LP, CB, and AH, Writing (original draft preparation): DS, OB, and LB, Writing (review and editing): OB, LB, DS and CC. Visualization: CB, RB, and AH. Supervision: AI and OB. Project administration: AI and OB. Funding acquisition: CC, CB, and AI. All authors contributed to the article and approved the submitted version.

## Funding

This research was funded by the grant Partnership for the transfer of knowledge in biogenomics applications in oncology and related fields-BIOGENONCO, Project co-financed by FEDR through Competitiveness Operational Programme 2014–2020 (contract No. 10/01.09.2016).

## Conflict of Interest

The authors declare that the research was conducted in the absence of any commercial or financial relationships that could be construed as a potential conflict of interest.
